# Pharmacometric modeling of lipid nanoparticle-encapsulated mRNA therapeutics and vaccines: A systematic review

**DOI:** 10.1016/j.omtn.2025.102686

**Published:** 2025-08-14

**Authors:** Miao Zhang, Linh Van, Mansoor M. Amiji

**Affiliations:** 1 Moderna Therapeutics, 325 Binney Street, Cambridge, MA 02142, USA; 2 Department of Pharmaceutical Sciences, School of Pharmacy, Bouvé College of Health Sciences, Northeastern University, Boston, MA 02115, USA; 3 Department of Chemical Engineering, College of Engineering, Northeastern University, Boston, MA 02142, USA

**Keywords:** MT: Delivery Strategies, messenger RNA, mRNA, lipid nanoparticles, LNP, absorption, distribution, metabolism, and excretion, ADME, pharmacokinetics, PK, quantitative models, model-informed drug development, MIDD

## Abstract

Significant progress has been made in the development of lipid nanoparticle (LNP)-encapsulated messenger RNA (mRNA)-based modalities with numerous candidates entering clinical trials. These novel modalities require the application of innovative quantitative models to inform the development of mRNA-LNP-based candidates. To this end, we conducted a comprehensive search of registered clinical trials related to mRNA-based modalities on ClinicalTrials.gov, summarizing the current advancements of mRNA-LNP-based modalities and their expanding therapeutic applications. Also, we performed a thorough review of quantitative models related to mRNA-LNP-based modalities from PubMed, Google Scholar, and Embase databases, exploring the model structures employed to capture the *in vivo* processes of mRNA-LNP, along with their current applications. Between 2002 and October 28, 2024, around 189 clinical trials were registered on ClinicalTrials.gov, encompassing approximately 132 unique mRNA-based modalities targeting 18 disease areas. There are 15 studies that have published quantitative models supporting both the preclinical and clinical development of mRNA-LNP-based therapeutics. Detail regarding quantitative modeling of mRNA-LNP, especially absorption, distribution, metabolism, and excretion, as well as the activation processes of immune responses induced by mRNA-LNP-based vaccines are reviewed. Furthermore, we offer insights for future research related to mRNA-LNP-related models, aimed at enhancing predictive performance and facilitating the expedited advancement of mRNA-LNP-related clinical development.

## Graphical abstract

**Figure:**
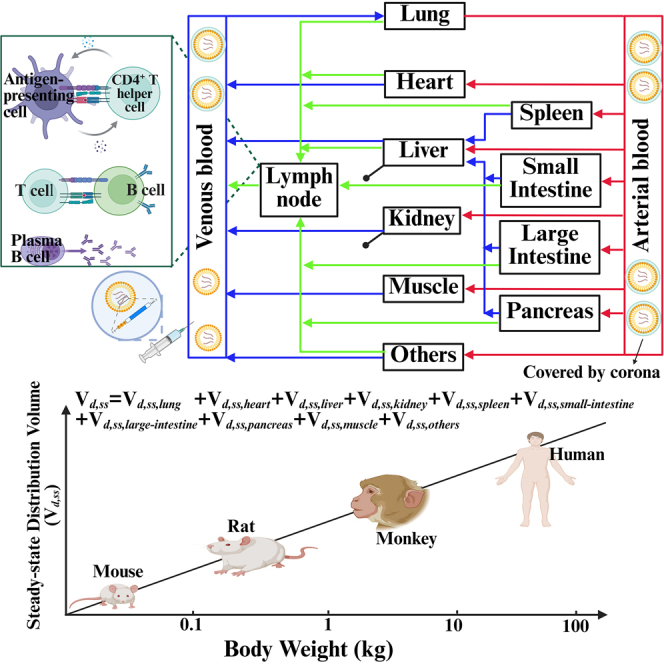

## Teaser

This review summarizes quantitative approaches for model-informed drug development of mRNA-lipid nanoparticle (LNP) modalities by incorporating absorption, distribution, metabolism, and excretion, pharmacokinetics/pharmacodynamics, and mechanistic models to guide first-in-human dosing and clinical study design. A literature review reveals that numerous clinical studies involving mRNA-LNPs have been conducted across a range of therapeutic areas and vaccine platforms.

## Introduction

Messenger RNA (mRNA)-based therapeutics harness the body’s cellular transcriptional and translational machinery to produce therapeutic proteins or antigens, eliciting potent pharmacological effects^1^ or triggering immune responses.^2^ This innovative approach offers a promising solution to a wide range of diseases, including those caused by protein insufficiencies,^1^ immune response deficiencies,^2^ and the need for targeted modulation of specific biological pathways.^3^^,^^4^ Unlike traditional protein-based drugs or gene therapy, mRNA-based modalities (referring exclusively to messenger RNA in this paper) directly instruct cells to produce desired proteins, enabling targeted and precision treatments.^1^ In recent years, mRNA therapeutics have demonstrated great promise in treating viral infections,^5^ cancer, and autoimmune diseases,^6^ with several therapies advancing through clinical development. Ongoing research continues to explore the potential of mRNA across an even broader array of medical conditions, underscoring its transformative role in modern medicine.

The first reported mRNA-based modality entered clinical trials in 2002, using dendritic cells to deliver mRNA for treating malignant melanoma (NCT01278940). Over the following decade, most clinical trials relied on dendritic cells for mRNA delivery, although none achieved market approval. A major challenge in mRNA-based therapeutics has been their inherent instability since mRNA is highly susceptible to degradation by nucleases in both the bloodstream and intracellularly. This issue was addressed in December 2015 with the introduction of a lipid nanoparticle (LNP)-encapsulated mRNA influenza vaccine encoding the H10N8 antigen (VAL-506440) into clinical trials (NCT03076385).^7^ The LNP formulation effectively shielded mRNA from premature degradation, enabling targeted delivery and enhancing its stability *in vivo*.^8^ This vaccine marked the first clinical trial utilizing the mRNA-LNP delivery system, overcoming critical barriers related to mRNA stability and delivery.

In addition to advancements in delivery technologies, quantitative approaches such as model-informed drug development (MIDD) have been pivotal in advancing mRNA-based therapeutics. The mechanism of action (MOA) of mRNA-based therapeutics involves several key steps, including delivery from the injection site to target tissues, cellular uptake, protein expression, and subsequent systemic distribution. MIDD enhances the understanding of these processes by building mathematical models to predict the *in vivo* behavior of the mRNA-based modalities.^9^^,^^10^ This approach bridges the species gap between preclinical models (e.g., mice, cynomolgus monkeys) and humans by aiding in the estimation of pharmacokinetic (PK) and pharmacodynamic (PD) characteristics. It also facilitates accurate dose extrapolation and rational dose design for both adult and pediatric populations.^4^^,^^6^^,^^11^^,^^12^

The structure of these quantitative models varies depending on the detectable concentrations of mRNA^11^ and the MOA of the encoded proteins,^6^^,^^12^ which can further add complexity to model development. For example, the *in vivo* process involving distribution and uptake of LNPs and mRNA to their translation of the encoded proteins is intricate, with many heterogeneities that can be challenging for even the development of mechanistic quantitative models. In response to these challenges, this review summarizes recent advancements in mRNA therapeutics, outlining the absorption, distribution, metabolism, and excretion (ADME) characteristics of therapeutic mRNA-LNP-based modalities, and examines the immune response activation process induced by mRNA-LNP-based vaccines, thereby providing a solid foundation for consideration during model development.

## Clinical development studies of mRNA-based modalities

**Figure 1:**
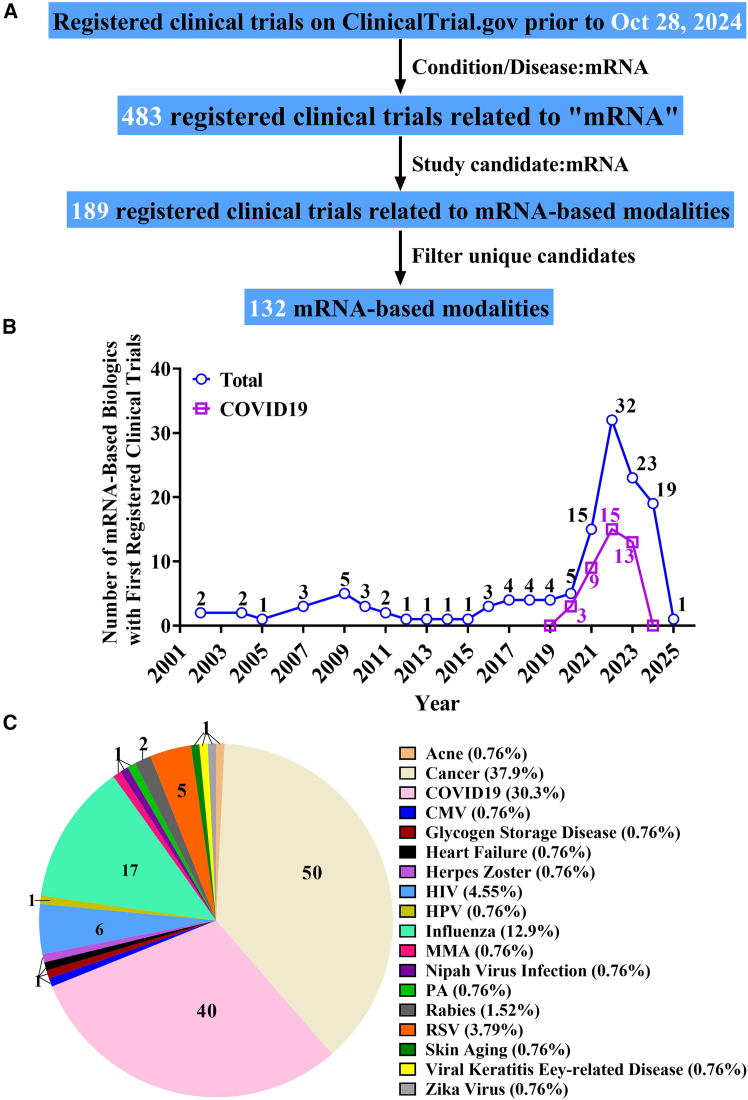
Clinical trial landscape of mRNA-based modalities (A Screening flowchart for identifying mRNA-based modalities on ClinicalTrials.gov. (B) Annual number of first-registered clinical trials from 2002 to 2024. (C) Number and proportion of mRNA-based modalities by disease type from 2002 to 2024. Note that all data are based on ClinicalTrials.gov as of October 28, 2024; includes one trial starting in 2025.

We conducted a search for registered clinical trials on ClinicalTrials.gov as of October 28, 2024, using the key word “mRNA” in the “Condition/Disease” field. A total of 483 clinical trials related to mRNA were identified. Of these, 189 clinical trials were associated with mRNA-based modalities, involving 132 unique candidates across the following study statuses: “Not yet recruiting” (11), “Recruiting” (35), “Active, not recruiting” (30), “Completed” (76), “Terminated” (15), “Enrolling by invitation” (1), “Suspended” (3), and “Unknown” (21). A flowchart illustrating this process is shown in Figure 1A, and a comprehensive summary of all candidates and their corresponding MOAs is included in Table S1. Based on our analysis, as illustrated in Figure 1B, the first clinical trial involving mRNA-based modalities was registered in 2002, marking the beginning of mRNA-based therapies entering clinical trials. In 2020, 5 mRNA-based modalities were registered for clinical trials, 3 of which were COVID-19-related vaccines. The development of mRNA-based modalities surged in 2021, with 15 candidates registering for clinical trials for the first time, including 9 of the COVID-19-related vaccines. The trend culminated in 2022, as 32 new candidates registered for clinical trials, comprising 15 COVID-19-related vaccines and 17 non-COVID-19 therapeutic candidates. Although the momentum of mRNA-based clinical research slowed after 2022, the number of non-COVID-19-related studies reached its peak in 2024 (as of October 28, 2024), with 19 mRNA-based modalities under development. Figure 1C presents the absolute numbers and corresponding percentages of mRNA-based modalities in clinical trials across various diseases. These mRNA drug candidates are associated with 18 major disease categories, with 38.2% focused on cancer-related diseases. Meanwhile, 30.5% target the prevention of SARS-CoV-2 infection, and 13.0% are designed to prevent influenza. Furthermore, mRNA-based modalities are being investigated for the prevention of other viral infections, including cytomegalovirus, herpes zoster, human immunodeficiency virus, human papillomavirus, Nipah virus, rabies, respiratory syncytial virus, and Zika virus. Other areas of exploration include treatments for conditions such as acne, skin aging, and heart failure, as well as metabolic disorders like methylmalonic acidemia and propionic acidemia. Collectively, these efforts aim to address a broad spectrum of unmet medical needs across diverse therapeutic areas.

## ADME properties of mRNA-LNP-based modalities

### PK characteristics of mRNA-LNP-based therapeutics

Depending on the routes of administration, the absorption of mRNA-LNP can be impacted by enzymatic degradation or limited permeability during gastrointestinal absorption. Intramuscular (IM), subcutaneous (SC), intradermal (i.d.) or intravenous (IV) injection, is commonly used for mRNA-LNP therapies. Following IM injection, particles smaller than 200 nm primarily traverse the interstitial space before entering lymphatic capillaries, where they are captured by regional lymph nodes.^13^ LNPs that bypass the lymph nodes subsequently enter the systemic circulation, constituting the “absorption phase” of mRNA-LNP formulation *in vivo*. SC injection follows an absorption process similar to that of IM injection, but it generally occurs more slowly. Once in the bloodstream, some LNPs may disassemble and release their mRNA payload, which is rapidly degraded by ribonucleases and eliminated. As a result, the kidneys play a minimal role in mRNA elimination. However, LNP encapsulation enhances mRNA stability, ensuring that its distribution *in vivo* is closely tied to the distribution of the LNPs. Upon entering the circulatory system, polyethylene glycol (PEG)-modified lipids desorb from the LNP surface, and plasma proteins effectively mask their original surface characteristics, creating a “protein corona.”^14^^,^^15^ This corona imparts a distinct biological signature to the LNPs, altering their interaction with target cells^15^^,^^16^ and modulating their biodistribution. Nonetheless, LNPs are transported via arterial circulation to organs with high blood flow, such as the lungs, liver, and spleen.^17^ In tissues, LNPs may cross endothelial barriers^18^ or pass through transcellular pathways such as fenestrations^19^ to enter the interstitial space. In this microenvironment, LNPs may be recognized as foreign entities,^20^ potentially initiating an immune response that leads to their sequestration, degradation, and clearance by the mononuclear phagocyte system (MPS), particularly in the liver and spleen but also in the lungs and kidneys.^21^ This process represents a primary elimination pathway for LNPs. Renal filtration clearance may depend mainly on their hydrodynamic diameter, with particles smaller than 10 nm being eliminated through this pathway.^22^ Meanwhile, in the interstitial space, LNPs can also be internalized by epithelial cells, where they release their mRNA payload for subsequent protein translation. Importantly, some LNPs may enter the lymphatic circulation, eventually returning to the bloodstream,^23^ while others directly enter the venous system following tissue perfusion. Together, these processes facilitate the complete *in vivo* disposition of LNPs.

### PK characteristics of mRNA synthesized proteins

Endogenous mRNA, referred to as “messenger” RNA, carries the genetic instructions required for protein synthesis. For specific therapeutic purposes, exogenous mRNA encapsulated in the LNPs or other delivery vehicles^24^ is utilized to replace deficient proteins in patients,^1^ modulate cell behavior by expressing transcription,^6^ or instruct cells to synthesize novel proteins, such as antigens.^12^ The mRNA-LNP can be internalized by target cells through endocytosis or macropinocytosis,^25^ where it subsequently reaches the acidic milieu of the endosome. Within the endosomal compartment, the LNPs undergo degradation, releasing their mRNA payload.^25^ This free mRNA escapes from the endosome and translocates to the cytoplasm, where it binds to ribosomes. The ribosomes then use the mRNA sequence as a template to guide the assembly of amino acids into a polypeptide chain, which may undergo proper folding and post-translational modifications to acquire its functional conformation. Depending on its specific function, the nascent protein may perform its role within various intracellular compartments^26^ or be secreted into the extracellular space.^1^ Alternatively, the protein may be integrated into the cell membrane.^27^ In cases where the synthesized protein is an antigen for a specific pathogen, it may be presented on the surface of antigen-presenting cells (APCs), initiating a cascade of immune responses. Therefore, the PK characteristics of these synthesized proteins should be understood based on their location.

#### ADME for intracellular proteins

Once synthesized, some proteins remain as permanent residents in the cytoplasm, while others are directed to specific subcellular compartment (e.g., nucleus, mitochondria, Golgi apparatus, endoplasmic reticulum). This localization is guided by sorting signals that instruct their transport to the appropriate destinations, constituting the micro-distribution process of these proteins *in vivo*.^28^ After fulfilling their functions or becoming damaged, intracellular proteins are typically tagged for degradation through the ubiquitin-proteasome pathway.^29^^,^^30^ In contrast, some misfolded or damaged intracellular proteins^31^ are generally internalized via endocytosis and directed to the lysosomes^32^ for degradation. The resulting degradation products can either be recycled within the cell or further processed.^33^ Additionally, peptides released into the extracellular space may be eliminated by the kidneys or cleared through the lymphatic system.^32^

#### ADME for membrane and extracellular proteins

After processing by the Golgi apparatus, modified proteins are encapsulated in vesicles, which either integrate them into the cell membrane or secrete them into the interstitial space or bloodstream.^34^ Proteins entering the interstitial space can be taken up by the lymphatic system, where they are transported to the lymph nodes before eventually returning to the bloodstream.^35^ Alternatively, some proteins are secreted directly into local blood vessels, from which they enter the venous circulation for systemic distribution.^36^ As these proteins circulate through the bloodstream, their concentration gradually increases, exhibiting an absorption-like phase. This process ensures the delivery of extracellular proteins to their target tissues but also contributes to the time lag between dose administration and detectable protein levels.^37^ When membrane-bound or extracellular proteins are no longer needed or become damaged, they are internalized by endocytosis and degraded within the lysosomes.^32^^,^^38^ In the lysosomal compartment, proteins are broken down into smaller peptides or amino acids, which may subsequently be filtered and excreted by the kidneys.^39^ For proteins with a molecular weight below 60 kDa, renal excretion represents an important elimination route.^40^ Furthermore, proteins circulating in the plasma or the lymphatic system^41^ may be recognized as foreign entities by the MPS, leading to their clearance by the liver or spleen. Collectively, these pathways contribute to the systemic clearance of the encoded proteins, representing a distinct renal elimination process compared to that of LNPs and mRNA.

### Factors impacting the ADME of mRNA-LNP-based therapeutics

#### Composition of LNPs

Current US Food and Drug Administration-approved LNP formulations typically consist of four primary lipid classes, each contributing uniquely to the efficiency and specificity of mRNA delivery and influencing various aspects of ADME. These lipid classes are as follows: (1) Ionizable lipids: ionizable lipids, such as Dlin-MC2-DMA, Dlin-MC3-DMA, and lipid 5,^42^ maintain a neutral charge at physiological pH, but undergo protonation in the acidic environment of endosomes.^43^ This process destabilizes endosomal membranes, enabling the release of mRNA cargo into the cytoplasm for subsequent protein synthesis.^44^^,^^45^ Additionally, the composition, steric structure, and pK_a_^46^ of ionizable lipids influence both tissue-specific mRNA expression^47^ and the retention of mRNA-LNP *in vivo*.^48^ (2) PEG-modified lipids: these lipids enhance the colloidal stability of LNPs by preventing aggregation,^49^^,^^50^ controlling LNP size,^51^ and providing a “stealth effect” that shields the LNP from immune system recognition.^52^ This modification reduces protein adsorption and opsonization on the LNP surface,^53^ limiting macrophage uptake, thereby increasing bioavailability and prolonging circulation time.^50^ (3) Cholesterol: this essential component contributes to the structural stability, integrity, and fusion of the LNPs, ensuring effective mRNA release. It also promotes tissue delivery^54^ and extends circulation half-life by reducing surface-bound proteins interactions.^55^ (4) Helper lipids: amphipathic phospholipids, such as DOPE (1,2-dioleoyl-*sn*-glycero-3-phosphoethanolamine)^56^ and DSPC (1,2-distearoyl-*sn*-glycero-3-phosphocholine),^42^ are commonly used as helper lipids to facilitate membrane fusion.^57^^,^^58^ These lipids can also affect LNP-protein interactions in the bloodstream, thereby influencing tissue-specific distribution. For example, DOPE tends to accumulate in the liver, whereas DSPC has been shown to contribute to spleen targeting under specific conditions.^59^^,^^60^ In addition to these core lipid components, recent advancements have incorporated selective organ-targeting molecules,^15^ which can modulate the overall pK_a_ and surface protein absorption of the LNP, thereby enabling organ-specific delivery. Furthermore, surface engineering strategies, such as ligand modification, have been employed to achieve targeted delivery of LNPs to specific locations within the body.^61^

#### Protein corona

The composition of the protein corona is crucial in determining the biological fate of LNPs, including their cellular internalization,^62^ targeting efficacy,^63^ and *in vivo* distribution,^15^^,^^62^^,^^63^^,^^64^ all of which are closely related to the ADME properties of LNPs. The protein corona can be classified based on the physiological functions, with major components including human serum albumin, apolipoproteins, immunoglobulins, fibrinogen, complement proteins, acute-phase proteins, coagulation factors, tissue leakage proteins, and other plasma or cellular components.^65^^,^^66^^,^^67^ Human serum albumin, the most abundant plasma protein (∼40% of the total plasma proteins), typically makes up only 5%–6% of the protein corona.^68^ Upon binding to the surface of nanocarriers, albumin undergoes structural alterations, enabling it to act as a ligand for macrophage scavenger receptors,^69^ such as class A1, thereby contributing to the clearance of LNPs.^56^^,^^70^ Apolipoproteins, accounting for approximately 17%–25% of the total protein content,^65^^,^^66^ have been reported in the literature as the major proteins of the protein corona, wherein apolipoprotein E (ApoE) is primarily metabolized by hepatocytes and plays an essential role in the clearance and uptake of lipoproteins by the liver.^71^ Due to the structural and physicochemical similarities between LNPs and endogenous lipoproteins, such as the constitution, size, and density,^53^ ApoE bound to the surface of LNPs can function as an endogenous targeting ligand. This interaction facilitates the uptake of LNPs by hepatocytes through ApoE-mediated pathways. Additionally, immunoglobulins, complement proteins, and acute-phase proteins as opsonins, promote interactions between LNPs and tissue-resident macrophages, leading to rapid blood clearance and liver accumulation of LNPs through engagement with Fc and scavenger receptors expressed on the surface of macrophages.^72^^,^^73^^,^^74^ While fibrinogen, coagulation factors, and tissue leakage proteins are generally not classified as opsonins, they can modulate immune responses^75^^,^^76^ and thus influence LNP biodistribution. Furthermore, LNPs enriched in β2-glycoprotein I are preferentially targeted to the spleen, while those enriched in vitronectin are primarily directed to the lung.^15^

#### Physicochemical properties

Organs such as liver, spleen, and kidneys contain endothelial cells with unique fenestrations (pores), allowing for rapid filtration of substrates based on size and chemical charge. The average diameter of fenestrations in liver sinusoidal endothelial cells is approximately 107 nm, ranging from 103 to 300 nm in humans.^19^^,^^77^ LNPs within the 60- to 100-nm size range can pass through these fenestrations into the space of the perisinusoidal,^78^^,^^79^ where they may be taken up by hepatocytes. In the spleen, endothelial cells measure around 7.5 μm in length, with a caliber of 0.81 μm,^80^ making it more accessible for large LNPs over 200 nm.^81^ In contrast, the glomerular filtration apparatus in the kidneys has a size cutoff of around 10 nm, contributing to the rapid renal clearance of small NPs.^81^ Particle size also influences protein absorption on the LNP surface, including the type and abundance, which potentially affects tissue targeting.^82^ Furthermore, the pK_a_ of LNPs, which governs the surface change of LNP at different pH levels, is another critical factor impacting their distribution. LNPs with an apparent pK_a_ between 6 and 7 are more likely to target the liver, while those with a pK_a_ greater than 9 tend to target the lungs. Conversely, LNPs with a pK_a_ between 2 and 6 are better suited for spleen targeting.^15^ Deviations outside this narrow pK_a_ range may impair their ability to effectively deliver the payload.^83^

#### Physiological and other factors

Physiological factors such as age, sex, and body weight are commonly explored as potential covariates during the development of quantitative models for mRNA-LNP-based modalities. These factors may potentially influence both PK and PD behaviors by modulating physiological characteristics such as organ blood flow, receptor expression, vascular permeability, and immune status, all of which may affect LNP distribution, cellular uptake, and systemic clearance. For example, liver sinusoidal endothelial cells in neonatal rats exhibit a strong filtration effect on particles larger than 250 nm in diameter,^83^ whereas the average fenestration diameter in normal adult rats is about 197 nm,^84^ potentially influencing LNP access to hepatocytes. Similarly, age-dependent differences in lipoprotein profiles have been observed: serum ApoE levels in pediatric subjects (3.8 years old) range from 2.64 to 4.72 mg/dL,^85^ compared to 3–7 mg/dL in the plasma of normolipidemic adults.^86^ Since ApoE mediates LNP targeting primarily via the low-density lipoprotein receptor (LDLR) pathway,^87^ such ontogenetic differences may influence ADME characteristic of LNPs. Notably, LDLR expression is significantly higher in healthy adults than in elderly patients,^88^^,^^89^ indicating that age-related changes in receptor abundance could further impact the tissue-specific distribution of LNPs. Moreover, the location of receptors may influence LNP distribution, as NP accumulation in tissues tends to align with LDLR expression levels.^90^ Sex-based differences also merit consideration, as sex-specific protein coronas exist and cause variations in preclinical immune responses.^91^^,^^92^

## Advancements in quantitative models for mRNA-LNP-based modalities

We conducted a review of 15 published papers on quantitative models for mRNA-LNP-based modalities using PubMed, Google Scholar, and Embase databases with a cutoff date of November 3, 2024. The search was based on key words such as “mRNA-LNP,” “PK/PD model,” “IS/ID model,” “KPD model,” “PBPK model,” and “QSP model.” Detailed information from these studies is summarized in Table 1. Currently, the immunostimulatory/immunodynamic (IS/ID) model developed for mRNA-1273 is the only model employed for dosing optimization in the pediatric population during clinical stages. In contrast, the other models primarily focus on supporting preclinical dose regimen design, demonstrating candidates’ efficacy in animal studies, and extrapolating doses from animal species to humans in the first-in-human (FIH) studies. Collectively, these models contribute to the development of both mRNA encoded therapeutic proteins and mRNA-related vaccines.

**Table 1:** Summary of quantitative models developed for mRNA-LNP-based modalities supporting both preclinical and clinical investigations

No.	Drug names	Model types	Stage	Institutes	Species	Software	References
1	AZD8601	PK/PD	preclinical	AstraZeneca	mice	Phoenix	Almquist et al.^9^
2	hPBGD mRNA	PK/PD	FIH	University of Navarra/Moderna	mice/rats/rabbits/monkeys	NONMEM	Parra-Guillen et al.^93^
3	FGF21	PK/PD	preclinical	AstraZeneca	mice	Phoenix	Bartesaghi et al.^10^
4	mRNA-3927	PK/PD	FIH	Moderna	mice/rats/monkeys	Pumas	Attarwala et al.^11^
5	mRNA-0184	PK/PD	FIH	Moderna	monkeys	Phoenix	Kaushal et al.^4^
6	mRNA-6231	KPD	FIH	Moderna	monkeys	Pumas	Liric Rajlic et al.^6^
7	HIV related mRNA	KPD	preclinical	Moderna	mice	Phoenix	Narayanan et al.^3^
8	IM-injected LNPs	PBPK	preclinical	Shanghai Jiao Tong University/Purdue University	mice	MATLAB SimBiology	Di et al.^13^
9	mRNA encoded luciferase	PBPK	preclinical	University of Pennsylvania	mice	ADAPT 5	Parhiz et al.^37^
10	hUGT1A1-modRNA	QSP	FIH	Applied BioMath/Alexion Pharmaceuticals	rats	MATLAB	Apgar et al.^26^
11	hUGT1A1-modRNA	minimal PBPK-QSP	preclinical	Moderna	rats	MATLAB SimBiology	Miyazawa et al.^94^
12	CRISPR-Cas	QSP	FIH	University of Florida	mice/monkeys/humans	MATLAB SimBiology	Desai et al.^95^
13	RSV pre-F mRNA/LNP	PK/PD	preclinical	Merck	mice	NONMEM	Austin et al.^96^
14	mRNA-1273	IS/ID	clinical	Moderna	humans	Pumas	Ivaturi et al.^12^
15	mRNA vaccine	QSP	–	University of Trento	–	–	Selvaggio et al.^97^

### PK/PD models for therapeutic mRNA-LNP-based therapeutics

**Figure 2:**
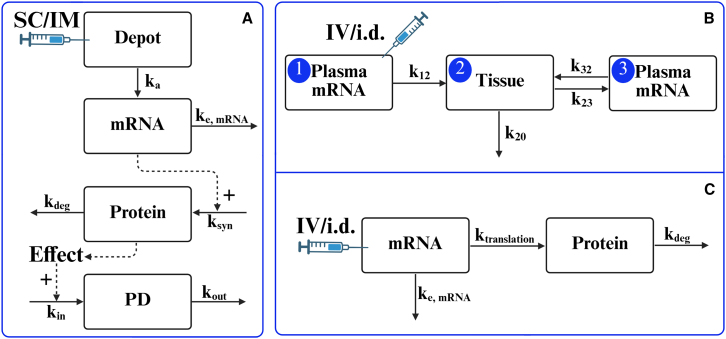
Overview of PK/PD model structures for mRNA-LNP-based modalities (A) Basic PK/PD model structure for SC/IM administration. (B) PK model with mRNA distribution for IV/i.d. administration. (C) Alternative model structure describing protein translated following IV/i.d. administration.

The development of PK/PD models for mRNA-LNP requires slight adaptation to capture the PK of the LNP/mRNA with the translation of the protein that is associated with the PD response. Depending on the route of administration, a depot compartment is used to represent the absorption process for SC and IM injections (Figure 2A). Meanwhile, the PK model structure is further adapted based on the detectability of mRNA concentrations in the bloodstream and the rate at which newly synthesized protein is distributed into systemic circulation. Due to the instability of mRNA in the circulation, mRNA concentration typically cannot serve as the primary PK indicator. Nonetheless, the mRNA-related compartment is retained in the PK model to account for the potential dynamic process of exogenous mRNA. Such models are sometimes referred to as kinetic-PD (KPD) models. However, in some cases, mRNA concentrations may be available to support the assessment of PK characteristics. A more complex semi-mechanistic model is then employed to capture the unique distribution dynamics of mRNA (Figure 2B), including redistribution from tissue to plasma to explain the biphasic nature of the mRNA time-concentration profile.^11^ In this model structure, a new plasma compartment is introduced to distinguish this redistribution process. This behavior is analogous to that observed with small interfering RNA-based therapeutics, such as patisitran,^98^ which also exhibits a secondary concentration peak due to tissue-to-plasma redistribution. Since mRNA-encoded proteins act as key intermediaries between dose and efficacy, a protein compartment is included in the model to describe both protein synthesis and degradation. For mRNA-encoded extracellular proteins, there is typically a spatiotemporal disconnect between dose administration and the appearance of the newly expressed protein. The protein compartment in Figure 2A provides a means to capture the time- and location-dependent behavior of such proteins.^4^^,^^6^^,^^11^^,^^93^ However, certain proteins can either be rapidly translated and distributed systemically or exert their function intracellularly.^3^ As illustrated in Figure 2C, this alternative model structure allows for the representation of such rapid dynamic processes. Because protein concentration can also function as a PK indicator in therapeutic mRNA-LNP-based modalities, the protein compartment is generally placed within the PK section of the model and may incorporate a peripheral compartment to describe its distribution dynamics in systemic circulation. The development of the PD model for therapeutic mRNA-LNP-based modalities parallels that of conventional protein therapeutics, using mathematical frameworks to define the relationship between the mRNA-encoded protein and its associated biological effects. Thus, the distinctive feature of PK/PD models for the therapeutic mRNA-LNP-based modalities lies in their PK model structure.

### Comprehensive insights into the physiologically based PK/quantitative systems pharmacology models for therapeutic mRNA-LNP-based modalities

Although there is still ongoing research into the *in vivo* mechanisms of mRNA-LNP, models developed for mRNA-LNP-based modalities are progressively integrating more mechanistic details. For instance, the key kinetic processes of mRNA-LNP formulation at the injection site following IM administration^13^ have been included in a minimal physiologically based PK (PBPK) model. This model is the first to define the bioavailable dose as the quantity of mRNA-LNP absorbed by the lymphatic vessels, offering valuable insights into the absorption phase after IM administration. The tendency of LNPs to accumulate in high-blood-flow organs, such as the lungs, heart, liver, kidneys, and spleen, has been demonstrated by experimental results using radioactive isotope-labeled LNPs and mRNA-encoded luciferase protein. This phenomenon has been incorporated into a comprehensive PBPK framework for LNP-encapsulated mRNA.^37^ Meanwhile, the model captures the spatiotemporal disconnect between tissue uptake of LNPs and protein expression from exogeneous mRNA. Furthermore, the detailed *in vivo* dynamics of mRNA-LNP have been modeled by various authors,^94^^,^^95^ including interactions between plasma proteins and LNPs, dissociation of the protein corona, receptor-mediated binding and unbinding of the LNP-protein corona complex, micropinocytosis through paracellular pores driven by lymph flow convection, and their degradation throughout the circulation.

### Summary of current modeling approaches for mRNA-LNP-based vaccines

The *in vivo* process for proteins initiating immune response for mRNA-LNP-based vaccines is designed to encode specific antigens that elicit adaptive immune responses. As a result, the focus shifts from traditional ADME characteristics to immune activation mechanisms. After being taken up by APCs, the mRNA is translated into the target antigen within the cytoplasm.^99^^,^^100^ These newly synthesized antigens are degraded by the proteasome and subsequently processed in the endoplasmic reticulum, where they are loaded onto major histocompatibility complex (MHC) class I molecules.^99^ The resulting MHC class I-peptide complexes are then presented on the cell surface,^32^ initiating a cytotoxic immune response mediated by CD8^+^ T cells. In addition to MHC class I processing, some nascent secreted antigens can be recognized and internalized by APCs,^99^^,^^101^ triggering the MHC class II presentation pathway. In this case, the antigens are processed into peptide fragments within the endolysosomal compartments. These degradation products bind to the MHC class II molecules, forming MHC class II-peptide complexes that are displayed on the surface of APCs. This presentation is recognized by CD4^+^ helper T cells,^102^ a critical step in activating the adaptive immune system.

Upon activation, helper T cells differentiate into both memory T cells and functional subsets. Among these, the T helper type 2 cells secrete cytokines that stimulate naive B cells, driving their differentiation into activated B cells.^103^ Subsequently, these activated B cells further differentiate into short-lived plasma cells, long-lived plasma cells, and memory B cells.^104^ Plasma cells are the primary contributors to antibody production in the adaptive immune response. Notably, activated B cells can also secrete antibodies, although their contribution is relatively minor compared to that of plasma cells. In parallel, both memory T and B cells remain in a dormant state but are primed to mount a rapid and robust response upon re-exposure to the same pathogen,^105^ thereby ensuring long-term immunity. To reinforce this protection, vaccines are typically administered in multiple doses. Booster doses not only enhance the adaptive immune response but they also stimulate memory B cells to proliferate and differentiate into plasma cells, increasing the production of pathogen-specific antibodies.^106^ Additionally, memory T cells are reactivated upon boosting, contributing to a faster and more coordinated cellular immune response. Over time, plasma cells undergo turnover, gradually diminishing in number, and the antibodies they produce are cleared from the body.^107^ However, both memory T and B cells persist in lymphoid tissues, maintaining enduring protection by remaining ready to respond swiftly to future pathogen encounters.^108^ This ongoing cycle signifies the resolution of the immune response and the establishment of long-term immunity.

**Figure 3:**
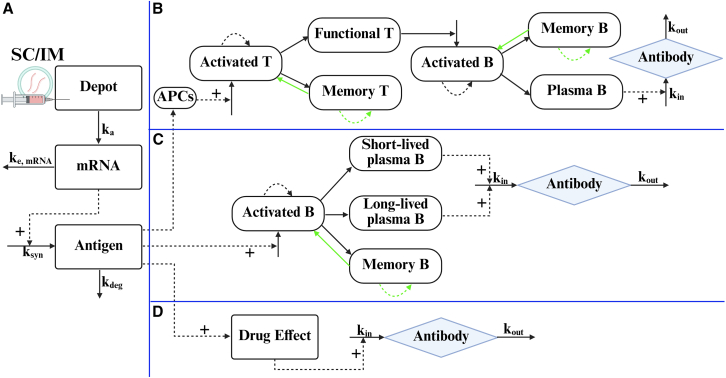
Schematic of the immune response models for mRNA-encoded antigens (A) PK-related compartments. (B) Comprehensive immune response modeling incorporating both T and B cell dynamics. (C) Simplified immune response model including only B cell dynamics. (D) Simplest model representing dose-mediated immune response. Curved dashed arrows represent clonal expansion; straight-dashed and right-angled dashed arrows denote PD effects; the green arrow indicates dynamic processes following a booster dose.

Currently, approximately 50% of mRNA-based modalities are designed for the prevention of viral infections, yet only three quantitative models have been developed specifically for mRNA-LNP-based vaccines to date. Figure 3 summarizes these model structures: panel A illustrates a general compartmental model for mRNA-encoded antigens, while panels B–D show progressively simplified representations of the immune response. Building on the MOA of antigen-induced immune responses, a quantitative systems pharmacology (QSP) model tailored for mRNA-LNP-based vaccines^97^ has been developed that incorporates APCs, T cells, and B cell-mediated immune responses (Figure 3B). The model enables the prediction of immune memory formation and antibody kinetics following mRNA vaccine administration, providing valuable insights into vaccine efficacy. In addition to the QSP model, an IS/ID model has been proposed (Figure 3C), focusing exclusively on the adaptive immune response mediated by B cell populations.^12^ This model tracks antibody levels over time and offers a simpler alternative to the QSP model. Another example is the KPD model,^96^ which uses a single “drug effect” compartment (Figure 3D) to represent the complex adaptive immune response. Although it is one of the simplest approaches, its ability to capture immune response dynamics has been validated by data from mice.

## Application of quantitative models in supporting preclinical and clinical development of mRNA-LNP-based candidates

### Enhancing preclinical development of mRNA-LNP-based modalities through MIDD

Quantitative PK/PD and mechanistic models are routinely being used in the early stages of developing mRNA-LNP-based modalities. Almquist et al.^9^ and Bartesaghi et al.^10^ established PK/PD models to quantify the mRNA-LNP dose required to achieve the half-maximal effective concentration effect, helping identify the optimal dose for treatment and support efficacy outcomes. Similarly, Austin et al.^.96^ applied the KPD model to demonstrate that split-dosing regimens can generate antibody responses comparable to, or even exceeding, those achieved by traditional bolus immunization methods.

### MIDD facilitates dose extrapolation for mRNA-LNP-based therapies in FIH studies

The impact of the protein corona on the *in vivo* fate of LNPs, coupled with variability in protein expression efficiency, makes effective dose extrapolation for mRNA-LNP-based therapies highly dependent on preclinical species data. To address species-related challenges, numerous studies have combined allometric scaling with PK/PD models, utilizing body weight-based allometric exponents to extrapolate critical parameters from animal studies to humans^1^^,^^3^^,^^4^^,^^6^^,^^11^^,^^93^ for subsequent estimation of systemic exposure and the selection of starting doses for FIH studies. Table 2 summarizes the findings from the literature, demonstrating that the allometric principle holds true across various species and modalities, including mRNA-LNP-based modalities. This is supported by the mean and median values of the allometric exponents, which exhibit small deviations from the typical values of 0.75 for clearance and 1.0 for volume. The standard deviations and coefficients of variation from across modalities are also low, indicating limited variability. Furthermore, by incorporating physiological factors and population variability, PBPK/QSP models facilitate the mechanistic translation of preclinical findings for mRNA-LNP therapies to humans.^26^^,^^95^ This capability is particularly valuable for mRNA-LNP-based modalities, in which mRNA and/or protein concentrations may be undetectable in systemic circulation, providing an accessible and reliable pathway for understanding *in vivo* dynamics.

**Table 2:** Comparison of the weight-based allometric exponent among mRNA-LNP, small molecules, and biologics

Drug types	Comments	mRNA, small molecules, and biologics				Translated proteins			Species	References
		k_a_	CL	Q	V	K_trans_	CL	V		
mRNA-LNP	mRNA-6231	−0.25	0.65	–	1	−0.25	0.85	1	monkeys and humans	Liric Rajlic et al.^6^
	mRNA-0184	–	0.75	–	1	–	0.85	1	monkeys and humans	Kaushal et al.^4^
	mRNA-3927	–	1.1	0.63	1	–	–	–	monkeys, mice, rats, and humans	Attarwala et al.^11^
	mRNA-3705/mRNA-3927/mRNA −3210	–	0.75	–	1	–	–	–	monkeys, mice, rats, and humans	Baek et al.^1^
	–	–	0.75	–	1	0.8	0.93	1	mice and humans	Narayanan et al.^3^
Small molecules	creatine clearance	–	0.68	–	–	–	–	–	14 species	Edwards^109^
	inulin clearance	–	0.72	–	–	–	–	–	26 species	Edwards^109^
	85 drug molecules	–	0.87	–	0.99	–	–	–	multiple species	Huang et al.^110^
	prediction using ≥2 species	–	0.603	–	0.900	–	–	–	3 species	Mahmood and Balian^111^
		–	0.767	–	0.907	–	–	–	2 species	Mahmood and Balian^111^
	54 extensively metabolized drugs	–	0.66	–	–	–	–	–	rats and humans	Chiou et al.^112^
	free concentration with IV	–	0.799	–	–	–	–	–	–	Feng et al.^113^
	free concentration with oral administration (PO)	–	0.789	–	–	–	–	–	–	Feng et al.^113^
		–	0.67	–	0.93	–	–	–	rats and humans	Caldwell et al.^114^
	renal secretion	–	0.809	–	–	–	–	–	one preclinical species and humans	Mahmood^115^
	biliary excretion	–	0.938	–	–	–	–	–		Mahmood^115^
	IV	–	0.782	–	–	–	–	–		Mahmood^115^
	PO	–	0.664	–	–	–	–	–		Mahmood^115^
	macromolecules	–	0.787	–	–	–	–	–		Mahmood^115^
Biologics	mAbs targeting soluble antigens	–	0.85	–	–	–	–	–	monkeys and humans	Ling et al.^116^
	mAbs targeting membrane-bound antigens	–	0.9	–	–	–	–	–	monkeys and humans	Ling et al.^116^
	mAbs	–	0.8	0.75	1	–	–	–	monkeys and humans	Haraya et al.^117^
	recombinant human interferons	–	1.1	1.46	1.35	–	–	–	rats/monkeys/mice, and humans	Kagan et al.^118^
	ADC analyte	–	1	–	–	–	–	–	monkeys and humans	Li et al.^119^
	membrane-bound target	–	0.96	–	1	–	–	–	monkeys and humans	Dirks and Meibohm^120^
	soluble target	–	0.79	–	1.12	–	–	–	monkeys and humans	Dirks and Meibohm^120^
	–	–	0.851	–	–	–	–	–	monkeys and humans	Mahmood^121^
	–	–	0.874	–	–	–	–	–	3 preclinical species and humans	Mahmood^121^
Sample statistical results	mean (SD)	–	0.81 (0.13)	0.95 (0.45)	1.02 (0.12)	–	0.88 (0.05)	1.00 (0.00)	–	–
	median (min, max)	–	0.79 (0.603–1.1)	0.75 (0.63–1.46)	1.00 (0.9–1.35)	–	0.85 (0.85–0.93)	1.00 (1.00–1.00)	–	–
	N	–	27	3	12	–	3	3	–	–

ADC, antibody-drug conjugate; CL, clearance; ka, absorption rate; K_trans_, mRNA-to-protein translation rate; mAbs, monoclonal antibodies; PO, per os; Q,intercompartmental rate; SD, standard deviation; V, volume.

### MIDD enables dose optimization for mRNA-LNP-based vaccines during clinical development

The MIDD approach is also relevant in the clinical development of mRNA-LNP-based therapies. For example, Rhodes et al.^122^ developed a mathematical model, referred to as the IS/ID model, to understand the effect of IS by the vaccine booster on the ID of the antibody titer. This model identified optimized dosages for improving the therapeutic outcomes of COVID-19 vaccines and highlighted that age variability is a critical factor necessitating dose adjustments. Similarly, based on another IS/ID model established by Ivaturi et al.,^12^ it was concluded that a 25-μg primary vaccination dose of mRNA-1273 would meet the non-inferiority criteria for immunogenic response in young children and infants, a finding that was later validated by subsequent research. This MIDD approach, which guides dosing decisions for vaccine therapy, has provided valuable insights for diverse populations with varying disease states, ultimately enhancing the effectiveness of the protection offered by mRNA-LNP-based vaccines.

## Future perspectives and recommendations

The recent regulatory approval of several mRNA-LNP-based modalities has led to an increase in the number of mRNA-LNP-based candidates being pursued. mRNA-LNP-related technology provides distinctive advantages over the traditional development of small and large molecules by leveraging platform data that can advance the drug candidates faster to clinic with higher success rates. However, significant knowledge gaps persist in understanding the *in vivo* behavior of mRNA-LNP-based therapeutics, highlighting the need for ongoing efforts to refine and optimize the predictive performance of quantitative models for these modalities in both preclinical and clinical studies. Many of the key dynamic processes involved in the *in vivo* behavior of mRNA-LNPs, such as the rapid degradation of mRNA, the fraction of mRNA that escapes from endosomes, and the efficiency of protein translation, lack direct experimental measurements. As a result, model development often relies on prior assumptions whether leveraging from the literature or preclinical sources or adapting based on knowledge from similar platform modalities or underlying biological principles.^94^^,^^95^ Even with these additional inputs, such assumptions can introduce considerable uncertainty and may compromise both model stability and the reliability of parameter estimation, especially when sample sizes are small or datasets are sparsely collected. Moreover, the significant variability observed in mRNA and protein levels across individuals can introduce additional complexity that must be accounted for during model development. To improve model reliability and support confidence in the extrapolation across species or different clinical settings, it would be beneficial to have a larger sample set to reduce uncertainty in parameter estimation and support more robust model calibration. Furthermore, mechanistic studies are needed to better understand the fate of mRNA *in vivo*. This is particularly important for mRNA-LNP-based vaccines, where immune dynamics are central to efficacy, and only dosage and antibody levels are typically available for model development. Additionally, model complexity may need to be revised to a simpler identifiable approach to ensure robust precision of estimates with low shrinkage. Additionally, estimation may not be limited to classical methods such as first-order conditional estimation with interaction, alternative approaches such as the stochastic approximation expectation maximization algorithm or Bayesian estimation (e.g., maximum a posteriori) or even novel artificial intelligence (AI) algorithms^123^^,^^124^ (e.g., neural networks, support vector regression, random forests, extreme gradient boosting and light gradient boosting machine) may need to be considered to handle very complex high-dimensional models.

The allometric principle appears to be valid and applicable for mRNA-LNP therapies in dose extrapolation,^1^^,^^4^^,^^6^^,^^11^^,^^93^ yet the weight-based allometric exponent for mRNA-LNP still requires further attention. For example, exponents of 0.65 and 0.85 have been used to extrapolate the clearance of mRNA-6231 (undetectable in the bloodstream) and its encoded protein from monkeys to humans, respectively, with the predicted protein PK later validated well by FIH study data.^6^ However, for mRNA-3927 (detectable in the bloodstream), data from mice, rats, and monkeys revealed exponents of 0.63 for initial intercompartmental clearance and 1.1 for both redistribution intercompartment and systemic clearance.^11^ These variations in the allometric exponents are partly attributed to differences in scaling derived across species and their associated biological and metabolic differences, which have been observed in both the mRNA distribution and their protein translation (see Table 2). Although the initial selection of allometric exponents should be guided by well-established allometric principles, adjustments may be warranted based on prior assumptions as well as the empirical model performance. Nonetheless, these findings are consistent with previous reports that allometric exponents are not universal,^115^^,^^125^ a limitation that also applies to mRNA-LNP-based modalities. Moreover, drugs with different clearance mechanisms, such as glomerular filtration,^109^ biliary excretion,^115^ and hepatic metabolism^110^ or differing MOAs,^116^ may have distinct allometric exponents. Selecting an appropriate exponent for mRNA requires caution, as *in vivo* elimination of mRNA-LNP involves multiple pathways, including MPS-mediated LNP systemic clearance, protein corona-mediated targeting tissue uptake, and size-dependent organ clearance. Additionally, differences in the lipid compositions of LNPs may also be an important factor influencing the selection of these allometric exponents for mRNA.^93^^,^^126^

Although mechanistic whole-body PBPK model frameworks are continuously being proposed, further refinement is necessary, as critical aspects of mRNA-LNP distribution and elimination processes have not yet been fully integrated. Specifically, the influence of physicochemical properties, such as pK_a_,^15^ particle size,^127^ lipid composition,^128^ the extent of unsaturation in lipid chains,^129^ and the structure of lipid chains,^42^ on the *in vivo* distribution of mRNA-LNP has not been adequately considered. Given the diversity in protein corona types, the association and dissociation rates of LNPs with certain protein coronas remain uncertain, as well as how these rates vary across different populations, such as pediatric and elderly groups, and in various disease statuses. Additionally, the cellular uptake rates, tissue clearance rates, and protein expression rates of target molecule-modified LNPs differ significantly from those of unmodified LNPs,^37^ suggesting that mRNA-LNP PBPK models should be tailored to the specific composition of the LNP and the characteristics of the encapsulated mRNA. Other important factors warranting attention include the recycling mechanisms of LNPs and the degradation rates of mRNA in the bloodstream and various organs. These questions require further evaluation.

Considerable progress has been made in understanding the *in vivo* immune response to mRNA-LNP vaccines through quantitative models like the IS/ID and KPD models, but several challenges remain. For instance, incorporating complex immune responses into a mechanistic model requires a series of mathematical equations. However, insufficient experimental and clinical data make it difficult to accurately determine the parameters relevant to immune responses. Parameters such as immune cell proliferation, differentiation, and apoptosis rates can be quantified through *in vitro* experiments for specific vaccines, thereby reducing the number of parameters that need to be estimated and enhancing the reliability of model predictions. Additionally, the IS/ID model used for clinical dose selection did not account for individual variability in antibody clearance,^12^ a crucial factor influencing the estimation of vaccine efficacy across diverse patient populations. The scarcity of clinical data often necessitates compromises in estimating these parameters. To overcome these limitations, sampling the processing of the adaptive immune response offers a promising approach to improving feasibility and applicability for both preclinical and clinical data. Nevertheless, model development should be tailored to the characteristics of specific mRNA-LNP-based vaccines, and ongoing research is essential to advance model-informed mRNA-LNP-based vaccine development.

Recent advancements in machine learning, AI, and deep learning, along with their integration into PBPK, PK/PD, and QSP frameworks, have shown considerable promise in predicting systemic exposure and pharmacological behavior of novel therapeutic candidates.^130^^,^^131^^,^^132^ These approaches leverage empirical science by incorporating chemical structure information, biological target profiles, and other high-dimensional datasets into computational workflows to support the estimation of unknown parameters and the refinement of mechanistic models.^133^ Likewise, hybrid modeling strategies can also incorporate structural features of lipids and sequence characteristics of mRNA to inform intracellular mRNA kinetics and antigen-induced immune response dynamics, particularly during early stages of model development when experimental data are sparse. To further deepen mechanistic understanding, quantum chemical modeling offers atomistic insights into molecular interaction such as lipid-mRNA, mRNA-enzyme, and lipid-enzyme interactions,^134^ can be used to explore how molecular structures affect encapsulation efficiency, stability, endosomal escape, and biodistribution. The sophistication in AI algorithms, including supervised learning (e.g., neural networks, random forests), unsupervised learning (K-means and principal-component analysis), semi-supervised learning, and reinforcement learning (e.g., Q-learning, policy gradient methods), provide robust model optimization improvement.^135^ Altogether, these integrated and multi-scale modeling approaches present a powerful toolkit for advancing the design and development of mRNA-LNP therapeutics, providing the potential to improve predictive accuracy and support informed decision-making across both therapeutic and vaccine applications.

## Acknowledgments

M.Z. is supported by an Academic-Industrial Collaborative Postdoctoral Fellowship program between 10.13039/100019533Moderna Therapeutics, Inc., Cambridge, MA and 10.13039/100015257Northeastern University, Boston, MA.

## Author contributions

Conceptualization, M.Z., L.V., and M.M.A.; writing – original draft, M.Z.; writing – review & editing, M.Z., L.V., and M.M.A.; funding acquisition, M.M.A.; supervision, L.V. and M.M.A.

## Declaration of interests

L.V. is an employee and shareholder of Moderna.
